# Impact of delaying botulinum toxin treatment in patients with migraine during the COVID-19 pandemic

**DOI:** 10.1055/s-0043-1763490

**Published:** 2023-04-14

**Authors:** Henrique Nascimento, Gonçalo Videira, Sara Duarte, Carlos Correia, Carlos Andrade

**Affiliations:** 1 Centro Hospitalar Universitário do Porto, Hospital de Santo António, Serviço de Neurologia, Porto, Portugal.

**Keywords:** Migraine Disorders, Botulinum Toxins, COVID-19, Transtornos de Enxaqueca, Toxinas Botulínicas, COVID-19

## Abstract

**Background** Due to coronavirus disease 2019 (COVID-19) pandemic response measures, the administration of botulinum toxin (BTX) was delayed for many patients during the first lockdown period in Portugal.

**Objectives** To review the impact of postponing BTX treatment on migraine control.

**Methods** This was a retrospective, single-center study. Patients with chronic migraine who had done at least three previous BTX cycles and were considered responders were included. The patients were divided into two groups, one that has had their treatment delayed (group P), and one that has not (controls). The Phase III Research Evaluating Migraine Prophylaxis Therapy (PREEMPT) protocol was used. Migraine-related data were obtained at baseline and at three subsequent visits.

**Results** The present study included two groups, group P (n = 30; 47.0 ± 14.5 years; 27 females, interval baseline -1 ^st^ visit: 5.5 [4.1–5.8] months) and the control group (n = 6; 57.7 ± 13.2 years; 6 females; interval baseline–1 ^st^ visit 3.0 [3.0–3.2] months). No difference between the groups was present at baseline. When compared to baseline, the number of days/month with migraine (5 [3–6.2] vs. 8 [6–15] *p* < 0.001), days using triptans/month (2.5 [0–6] vs. 3 [0–8], *p* = 0.027) and intensity of pain (7 [5.8–10] vs. 9 [7–10], *p* = 0.012) were greater in the first visit for group P, while controls did not present a significant variation. The worsening of migraine-related indicators decreased in the following visits; however, even in the third visit, it had not returned to baseline. Correlations were significant between the delayed time to treatment and the increase in days/month with migraines at the first visit after lockdown (r = 0.507; *p* = 0.004).

**Conclusions** There was a deterioration of migraine control after postponed treatments, with a direct correlation between the worsening of symptoms and the number of months that the treatment was delayed.

## Resumo

**Antecedentes** Devido às medidas de resposta à pandemia de *coronavirus disease 2019* (covid-19), a administração de toxina botulínica (TXB) foi adiada para muitos pacientes durante o primeiro confinamento em Portugal.

**Objetivos** Avaliar o impacto do adiamento do tratamento com TXB no controle da enxaqueca.

**Métodos** Estudo retrospectivo unicêntrico. Foram incluídos pacientes com enxaqueca crônica com pelo menos três ciclos prévios de TXB e que tenham sido considerados respondedores. Os pacientes foram divididos em dois grupos, sendo um com atraso do tratamento (grupo P) e outro sem atraso (controles). O protocolo *Phase III Research Evaluating Migraine Prophylaxis Therapy* (PREEMPT) foi utilizado. Dados clínicos relacionados com a enxaqueca foram obtidos na consulta inicial (T0) e nas três consultas subsequentes (T1–3).

**Resultados** O presente estudo incluiu dois grupos, o grupo P (n = 30; 47,0 ± 14,5 anos; 27 mulheres, intervalo T0-1ª visita: 5,5 [4,1–5,8] meses) e o grupo controle (n = 6; 57,7 ± 13,2 anos; 6 mulheres; intervalo T0–1ª visita 3,0 [3,0–3,2] meses). Os grupos não apresentavam nenhuma diferença no início do estudo. Quando comparado à T0, o número de dias/mês com enxaqueca (5 [3–6,2] vs. 8 [6–15], *p* < 0,001), dias usando triptanos/mês (2,5 [0–6] vs. 3 [0–8], *p* = 0,027) e intensidade da dor (7 [5,8–10] vs. 9 [7–10], *p* = 0,012) foram maiores na primeira visita no grupo P, não apresentando os controles variação significativa. A piora dos indicadores relacionados com a enxaqueca diminuiu nas visitas seguintes; porém, mesmo na terceira visita, ainda não haviam retornado ao basal. As correlações foram significativas entre o atraso do tratamento e o aumento de dias/mês com enxaqueca na primeira consulta após o confinamento (r = 0,507; *p* = 0,004).

**Conclusão** Houve piora clínica da enxaqueca após o adiamento do tratamento em correlação direta com a duração do atraso.

## INTRODUCTION

Migraine is responsible for just under €100 billion euros of economic costs every year in Europe alone. The biggest part of this number is associated with loss in productivity (93%). 1 The impact of migraine in productivity is easy to understand considering that a large part of the affected patients is professionally active. 2

The way health care is delivered changed in the context of the coronavirus disease 2019 (COVID-19) pandemic, with a large number of medical appointments being either postponed or canceled. 1 Telemedicine was many times chosen over face-to-face appointments, particularly during lockdown periods. A study of the American Migraine Foundation (AMF) assessing patients' perspective of telemedicine for headache care during the pandemic (n = 1,098) reported a high percentage of satisfied patients with 82.8% reporting a very good or good experience, while only 3.6% reported a poor one. Moreover, 89.8% of patients indicated that they would prefer to continue to use telemedicine for their headache care. 3

Although non-presential appointments can be a good solution for non-acute headache management, they are not a valid option for individuals receiving botulinum toxin (BTX). This is particularly important as patients under BTX are usually the ones with more severe/refractory disease. 4 5

Migraine episodes are known to intensify during infectious intercurrences. Besides, severe acute respiratory syndrome coronavirus 2 (SARS-CoV2) infection itself is specifically associated with headaches, rendering it difficult to interpret the worsening of migraine in these patients. 1 Moreover, the restrictive measures and overall context of limitations associated with the pandemic has caused an increase in stress levels and mood disorders that could further influence and increase migraine severity. 6

Furthermore, after the SARS-CoV-2 infection is resolved, it is still necessary to worry about long COVID, a condition that is not fully understood. Headaches have been reported in up to 44% of patients with long COVID. 7 8

Due to governmental pandemic response measures, the administration of BTX was delayed for many patients during the first lockdown period in Portugal (March 16–May 11, 2020), a time when SARS-CoV-2 infection was poorly understood, and the focus was on preventing transmission. 9

In this paper, we review the impact of the delay of BTX treatment in migraine control. The main objective is to evaluate the variation in the number of headache days (including migraine-type) per month with treatment delay. The secondary objectives are to compare patients who had their treatment delayed with those whose therapeutic schedule was not impacted and to evaluate the time needed for migraine control to return to prepandemic levels after reintroduction of treatment.

## METHODS

This was a retrospective, single-center study of patients undergoing BTX infusion for the treatment of chronic migraine.

The inclusion criteria were patients with chronic migraine (≥ 15 days per month with headache lasting 4 hours a day or longer), who had undergone at least 3 previous BTX cycles and were considered responders (≥ 50% improvement from baseline). All patients received onabotulinumtoxin A (Botox, Allergan Pharmaceuticals Ireland, Westport, Ireland) injections according to the Phase III Research Evaluating Migraine Prophylaxis Therapy (PREEMPT) protocol. 10

The study was approved by the hospital ethics committee. All patients provided informed verbal consent.

Patients were divided into two groups, one that has had their treatment delayed for more than 2 weeks (group P) and one whose schedule remained unaltered (controls).

Clinical data regarding age, sex, number of days with headache (including migraine-type), pain intensity, days of disability per month, visits to the emergency department, response to treatment with BTX, concomitant prophylactic and acute treatment of migraine were collected from hospital clinical electronic platforms.

Baseline symptoms were considered at the last treatment before the interruption due to lockdown (March 16–May 11, 2020). 9 Baseline values were then compared to symptoms reported at the three visits that followed the restart of BTX application (2 BTX cycles, approximately 6 months after restart).

Statistical analysis was performed using the IBM SPSS Statistics for Windows, Version 26.0 (IBM Corp., Armonk, NY, USA). Normally distributed variables are presented as mean ± standard deviation; variables not normally distributed are presented as median (interquartile range). Comparisons between time periods were made using the Wilcoxon test. Spearman rank correlation was used for evaluating correlations between variables. Categorical variables distributions were compared with the χ ^2^ test. Statistical significance was defined by *p* < 0.05, using a two-sided test.

## RESULTS

Thirty-six patients fulfilled the inclusion criteria. Treatment delayed occurred in thirty of them (group P; 47.0 ± 14.5 years; 27 females, interval baseline-1 ^st^ visit: 5.5 [4.1–5.8] months), while only 6 patients maintained a regular follow-up (controls; 57.7 ± 13.2 years; 6 females; interval baseline-1 ^st^ visit 3.0 [3.0–3.2] months). No difference between groups was present at baseline ( Table 1 ).

**Table 1:** Comparison between migraine-related indicators in the last application of botulinum toxin before lockdown and in the three following medical appointments, according to having the treatment delayed or not

	Before lockdown	After lockdown		
		1 ^st^	2 ^nd^	3 ^rd^
**Patients whose treatment was not delayed (n = 6)**				
** Interval bs-1 ^st^ visit (months) **	3.0 (3.0–3.2)			
**Age (years)**	57.7 ± 13.2			
**Females**	6 (100%)			
**Days/month with headache**	9 (7–16.5)	9 (7.5–14)	9.5 (7.25–10.5)	8 (4.75–12)
**Days/month with migraine**	5.5 (3.75–9.5)	8.5 (2.25–9)	4 (2.25–9.75)	5.5 (1.5–9)
**Analgesics/month**	0 (0–2.75)	2.5 (0–4.5)	3 (0–8.5)	3.5 (2.25–4)
**Triptans/month**	2.5 (0–9.5)	4.5 (0–9.5)	2.5 (0–9.75)	0 (4–9)
**Pain intensity** (0–10)	8.5 (4.25–10)	9.5 (7.25–10)	8 (7–8.5)	8 (6.75–8.5)
**Days/month with incapacity**	1.17 (0–1.50)	0.66 (0–2.0)	0.83 (0–1.08)	0.33 (0–1.08)
**Visits to ED/month**	0	0	0	0
**Subjective improvement (vs. pre-botulinum toxin)**	50–75% 3 (50%) ≥ 75% 3 (50%)	50–75% 3 (50%) ≥ 75% 3 (50%)	50–75% 4 (66.7%) ≥ 75% 3 (33.3%)	50–75% 3 (50%) ≥ 75% 3 (50%)
**Patients whose treatment was delayed (n = 30)**				
** Interval bs-1 ^st^ visit (months) **	5.5 (4.1-5.8)$			
**Age (years)**	47.0 ± 14.5			
**Female**	27 (90%)			
**Days/month with headache**	8 (5–10)	12 (8–20.2)**	9.5 (8–13.5)^	8 (5–15)^
**Days/month with migraine**	5 (3–6.2)	8 (6–15)***	8 (6–11.2)***	7 (2–12)*
**Analgesics/month**	3.5 (0–6.5)	3 (0–15)^	3.5 (0–10)	4 (0–12)
**Triptans/month**	2.5 (0–6)	3 (0–8)*	4.5 (0–8)*	2 (0–7)
**Pain intensity** (0–10)	7 (5.8–10)	9 (7–10)*	9 (7–10)*	9 (8–10)*
**Days/month with incapacity**	0	0 (0–0.08)#	0 (0–0.74)^	0
**Visits to ED/month**	0	0	0	0
**Subjective improvement (vs. pre-botulinum toxin)**	50–75% 15 (50%) ≥ 75% 15 (50%)	< 25% 3 (10%)** 25–50% 3 (10%) 50–75% 15 (50%) ≥ 75% 9 (30%)	< 25% 3 (10%)* 25–50% 3 (10%) 50–75% 13 (43.3%) ≥ 75% 11 (36.7%)	25–50% 5 (16.7%)* 50–75% 13 (43.3%) ≥ 75% 12 (40%)

Abbreviations: bs, baseline; ED, emergency department.

Notes: *, ** and ***, *p* < 0.05, *p* < 0.01 and *p* < 0.001 vs. before lockdown in the same group, respectively. ^, *p* < 0.1 and > 0.05 vs. before stop in the same group. # and $, *p* < 0.05 and *p* < 0.001 vs controls, respectively.

When compared to the baseline, in the group P, not only the number of days with headache (8 (5–10) vs. 12 (8–20.2), *p* = 0.003) and with migraine (5 (3–6.2) vs. 8 (6–15) *p* < 0.001) increased, but also the number of days using triptans/month (2.5 [0–6] vs. 3 [0–8], *p* = 0.027) and the intensity of pain (7 [5.8–10] vs. 9 [7–10], *p* = 0.012) were greater in the first visit after the interruption. This worsening of migraine-related symptoms tended to decrease in the second and third visits after treatment restarted, but significant difference to baseline was only lost for days with headache and the number of days using triptans. Controls, on the other hand, did not present a significant variation during the studied period ( Table 1 ).

Despite the worsening of symptoms, no significant change in days with incapacity (vs. pre-BTX) and no visits to the emergency department were noticed ( Table 1 ).

**Figure 1:**
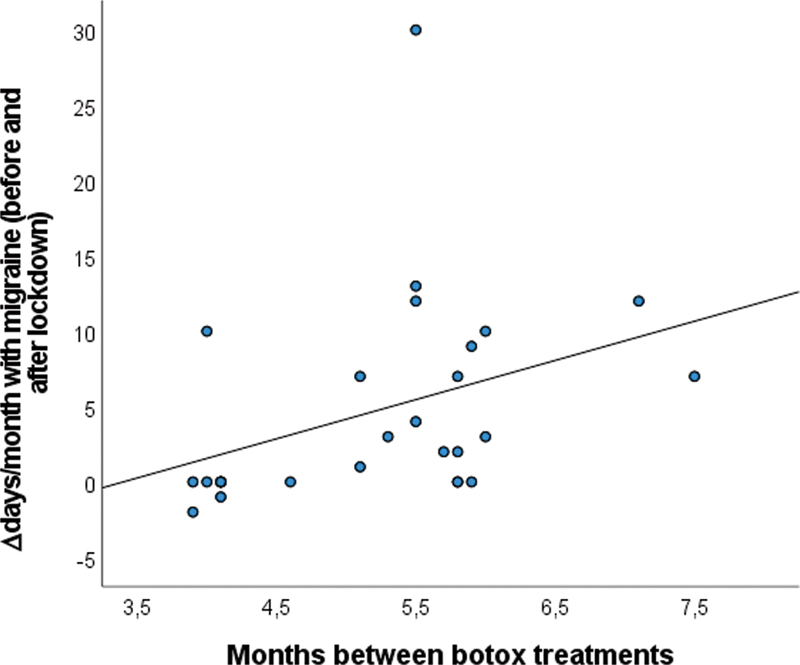
Correlation between the months of interruption of botulinum toxin treatment and the increase in migraines per month in the 1 ^st^ postlockdown appointment.

Spearman correlations were performed between the total months of interruption and the difference between baseline and the first visit values of headaches, migraines, analgesics, triptans, and pain intensity. There was a significant correlation between the number of months of treatment delayed and the difference in days with migraine (r = 0.507; *p* = 0.004; Figure 1 ) and the difference in days with headaches (r = 0.368; *p* = 0.045).

## DISCUSSION

The neurological services around the world had to adapt their routines during the COVID-19 pandemic, especially in lockdown periods. During the first lockdown in Portugal, BTX therapy was interrupted in our hospital for 2 months. Similarly, a study by Kristofferson et al. reported that only 36% of the neurological services in Denmark and Norway continued BTX treatment as usual, and 28% did not administer BTX at all during the lockdown period (1 month). As a result, some patients had longer-than-usual intervals between treatments (Denmark 25%, Norway 18%). 4 Longer intervals between treatments were frequent in many countries (e.g., Italy, 11 Spain, 12 13 and United States 14 ).

Our results show a deterioration of migraine control, with an increasing number of days with headache, days with migraine, use of triptans, and pain intensity in the patients whose treatments were postponed. On the other hand, patients who maintained treatment regularity kept a good response to BTX even in the face of a pandemic crisis. The importance of regular BTX cycles was further demonstrated in our population by the direct positive correlation between the frequency of headache and the duration of the delay in BTX administration.

Similar results were found by Porta-Etessam et al. in a small Spanish sample of 20 patients. In this population, after 1 month of delayed treatment, the mean days with headache per month increased from 9.5 ± 5.11 to 17.95 ± 8.94, and 75% of patients considered that they were overall worse. 13 Another Spanish study involving 67 patients showed that patients whose treatment was interrupted against their will (n = 9) presented 7 to 9 more days per month with headaches and migraine attacks compared to patients for whom delay was voluntary (n = 14) or did not occur (n = 44) during COVID-19 lockdown. Interestingly, no significant differences in the subjective worsening of migraine and the intensity of migraine attacks were found between groups. 12

On contrary, a Italian study evaluating the influence of a 2-month lockdown in 137 patients regularly treated with BTX for various conditions (94 cases and 43 controls; mean delay of treatment of 73.6 ± 26.5 days; migraine representing 10.63% and 11.62% of cases and controls, respectively) showed no difference in overall quality of life between cases and controls, even when different medical conditions were accounted for, and despite cases reporting subjective worsening comparing to controls. 11

Maintaining treatment regularity, particularly in patients with severe disease, such as those under BTX therapy, is crucial. When therapy is suspended, not only an abrupt worsening of symptoms is expected, but the return to the previous levels of response might take long. In agreement, the worsening of migraine-related indicators did not return to baseline levels even after 2 cycles of BTX injection in our population.

In conclusion, most patients understood the COVID-related contingencies and were willing to delay BTX treatment. However, with prolonged treatment interval came worsening of disease control, and individuals that were previously well saw their condition deteriorate in direct relation with the length of the delay. The impact of the temporary treatment suspension is not resolved easily with BTX reintroduction, as even after two cycles of BTX application (approximately 6 months) patients still have not returned to prelockdown values.

Therefore, it is important to keep a regular follow-up of migraine patients under BTX treatment. If that is not possible, it is important to consider newer therapeutic strategies that do not need face-to-face medical appointments, such as anti-calcitonin gene-related peptide (CGRP) antibodies. Evaluating comorbidities that influence migraine severity, such as depression, could help identify patients at greater risk of worsening with prolonged follow-up intervals.

This study has some limitations: first, it is a retrospective study based on self-reported calendars of symptoms and, thus, subject to memory biases; second, the number of patients involved is relatively small (although not much different from the other studies cited [12–14]); third, we did not evaluate potential confounders such as onset/worsening of comorbidities (e.g. depression) and concomitant SARS-Cov-2 infection, which could have influenced migraine severity.
